# CT and MRI Medical Image Fusion Using Noise-Removal and Contrast Enhancement Scheme with Convolutional Neural Network

**DOI:** 10.3390/e24030393

**Published:** 2022-03-11

**Authors:** Jameel Ahmed Bhutto, Lianfang Tian, Qiliang Du, Zhengzheng Sun, Lubin Yu, Muhammad Faizan Tahir

**Affiliations:** 1 School of Automation Science and Engineering, South China University and Technology, Guangzhou 510640, China; eejameelbhutto@mail.scut.edu.cn (J.A.B.); qldu@scut.edu.cn (Q.D.); ausunzz@mail.scut.edu.cn (Z.S.); 201610102119@mail.scut.edu.cn (L.Y.); 2 Southern Marine Science and Engineering Guangdong Laboratory Zhuhai, Zhuhai 519000, China; 3 Key Laboratory of Marine Environmental Survey Technology and Application, Ministry of Natural Resources, Guangzhou 510300, China; 4 School of Electric Power, South China University and Technology, Guangzhou 510640, China; epfaizantahir_2k7@mail.scut.edu.cn

**Keywords:** image fusion, medical imaging, healthcare, convolutional neural network

## Abstract

Medical image fusion (MIF) has received painstaking attention due to its diverse medical applications in response to accurately diagnosing clinical images. Numerous MIF methods have been proposed to date, but the fused image suffers from poor contrast, non-uniform illumination, noise presence, and improper fusion strategies, resulting in an inadequate sparse representation of significant features. This paper proposes the morphological preprocessing method to address the non-uniform illumination and noise by the bottom-hat–top-hat strategy. Then, grey-principal component analysis (grey-PCA) is used to transform RGB images into gray images that can preserve detailed features. After that, the local shift-invariant shearlet transform (LSIST) method decomposes the images into the low-pass (LP) and high-pass (HP) sub-bands, efficiently restoring all significant characteristics in various scales and directions. The HP sub-bands are fed to two branches of the Siamese convolutional neural network (CNN) by process of feature detection, initial segmentation, and consistency verification to effectively capture smooth edges, and textures. While the LP sub-bands are fused by employing local energy fusion using the averaging and selection mode to restore the energy information. The proposed method is validated by subjective and objective quality assessments. The subjective evaluation is conducted by a user case study in which twelve field specialists verified the superiority of the proposed method based on precise details, image contrast, noise in the fused image, and no loss of information. The supremacy of the proposed method is further justified by obtaining 0.6836 to 0.8794, 0.5234 to 0.6710, and 3.8501 to 8.7937 gain for $Q_{F}^{\mathrm{AB}}$, CRR, and AG and noise reduction from 0.3397 to 0.1209 over other methods for objective parameters.

## 1. Introduction

With the rapid evolution of computer technologies and sensors, medical imaging is playing an increasingly important role in various medical applications, including surgical navigation, clinical diagnosis, radiation surgery, and the treatment of serious diseases [1,2,3,4]. The medical images produced by different sensors focused on specific information such as tissues or bones in a body, but they cannot retain complementary information of both due to the limitations of sensors mechanism. For instance, computed tomography (CT) and magnetic resonance imaging (MRI) are used to diagnose various diseases such as brain tumors, head trauma, strokes, and so on [5,6,7,8], but CT focuses on bones and implants while MRI is limited to soft tissues. As a result, CT images miss soft tissue concerns, whereas MRI fails to capture bones and implants information.

**Figure 1:**
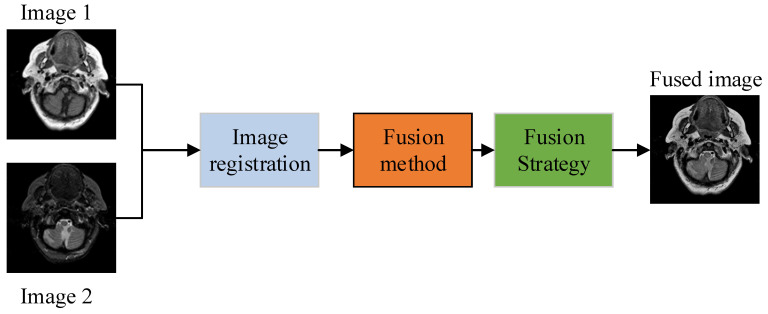
Basic block diagram of image fusion.

Due to the restricted information provided by a single sensor, doctors need to consume more time and effort to obtain complete information to diagnose the patient’s condition. Apart from this, the foremost information about a patient’s disease may be neglected due to collecting information from multiple imaging sensors, thereby reducing diagnosis accuracy. The effective way to tackle this problem is to fuse the multimodal images of a patient’s body from the same location to generate one image, which is known as medical image fusion (MIF). The MIF is a process in which information from different imaging sensors is merged to acquire one fused image that contains all unique characteristics. This not only helps doctors to save their time and energy but also can accurately diagnose various diseases such as brain tumors, trauma, and so on [9]. The image fusion method usually consists of three steps: load the images that are well-aligned and well-registered, apply a particular fusion method, then reconstruct to obtain one final fused image. Figure 1 shows the basic block diagram of any image fusion method [10].

The image fusion is acquired by three distinguished processing levels named pixel-level, feature-level, and decision-level [11]. Many researchers employ pixel-level-based fusion in a variety of applications. It directly merges the pixels of the input images to obtain the final output image. Feature-level-based image fusion on the other hand deals with high-level processing tasks [12,13]. It extracts the image features and then amalgamates them using advanced fusion schemes like region-based fusion. The decision level is the highest of the three processing steps described above. It extracts all information from images and then makes decisions to fuse the extracted features based on particular criteria [14].

The prime objective of any MIF method is to extract all features from multimodal sensors to produce one fused image that contains clear and precise detailed information from both images without generating noise. Over the last few decades, numerous authors have contemplated many attempts at MIF methods such as multi-scale decomposition (MSD)-based [15], neural network (NN)-based [16], deep CNN-based [1,3,17], sparse representation (SR)-based, and hybrid-based methods [18]. The MSD methods [10,19,20] produce good results for spectral information but lead to spatial information loss. On the other hand, the NN-based approaches [21] give good results, but the large number of tuning parameters set as constant by the experience of humans make them limited for specific applications. Many authors have used SR-based methods [22,23] for image fusion, but these methods cannot restore detailed information and have high sensitivity to misregistration. The CNN methods [24,25,26] have received remarkable attention in the last five years, but directly employing the CNN scheme in the spatial domain results in losing significant features. By keeping the aforementioned issues, many researchers have designed hybrid image fusion methods [27,28,29] to inherit the potential of multiple domains. Nonetheless, the fused image suffers due to non-uniform illumination, varying contrast, and improper fusion strategies. Additionally, the noise generated due to sensors, improper alignment and complex fusion strategies further distort the quality of fused image. In this context, this paper fills in the gaps of recent methods by proposing a novel hybrid MIF algorithm that alleviates the aforementioned shortcomings. To the best of our knowledge, this is the first attempt that proposed method uses the bottom-hat–top-hat strategy to remove the noise and uneven illumination. The bottom-hat operation is used to observe the effect of background noise, and then the top-hat operation is employed to remove noise and enhance varying contrast. Tt is well-known that gray-scale images preserve more detailed features than RGB images. Keeping this consideration, we have utilized grey-PCA that rotate the axes from intensity values of RGB space to three orthogonal axes to provide an effective RGB to gray conversion that preserve substantial features with high contrast. Additionally, it eliminates the redundant amount of data with its robust operation. After that, the LSIST is applied that decomposes the images into LP and HP sub-bands using non-subsampled pyramid filters (NSPF) and shearing filters (SFs) to efficiently restore the significant features in various scales and directions. The HP sub-bands are fused using two branches of Siamese CNN by process of feature detection, initial segmentation, and consistency verification to restore the smooth edges, textures and contours, while the LP sub-bands are fused by a local energy fusion strategy using averaging and selection mode by setting a threshold value to select the desired data. The proposed method surpasses recent fusion methods in terms of subjective as well as objective evaluation parameters.

The main contributions in our work are highlighted as:

- The morphological bottom-hat–top-hat is utilized to remove uneven illumination and noise by employing bottom-hat and top-hat operations. The bottom-hat operation is used to observe the noise effect and then the top-hat operation is used to remove the noise and enhance the contrast by subtracting the top-hat image from the bottom-hat image. Then, grey-PCA is used for conversion of the RGB image to a gray image, which preserves substantial features by increasing the contrast of the image.
- Then, the LSIST is applied that decomposes the images into LP and HP sub-bands by applying non-subsampled pyramid filters (NSPF) and local small-scale shearing filters (SFs) that efficiently restore salient features at distinguished scales and direction.
- The HP sub-bands are fed to two branches of the Siamese convolutional neural network (CNN) by three steps of feature detection, initial segmentation, and consistency verification to effectively capture smooth edges and textures, while the LP sub-bands are fused by employing local energy fusion using averaging and selection mode by setting threshold level to restore the desired energy information. At last, the inverse transformation is applied to fuse the low and high sub-images to produce a final output image containing substantial enriched details.

The remainder of the paper is arranged as follows: Section 2 elaborates the literature of existing MIF methods. Section 3 presents the discussion on our proposed work. The simulation results and their evaluation are explained in Section 4, and the conclusion with future work is stated in Section 5.

## 2. Literature Review

The MIF has played a crucial role due to its diverse medical applications such as brain tumors, trauma, radiotherapy, computer-aided medical diagnosis, and navigation of severe disease. Many researchers have attempted to improve the quality of MIF, and it has helped doctors to diagnose the disease accurately and saves their time and energy. Over the last few decades, numerous authors have contemplated many attempts at MIF methods such as multi-scale decomposition (MSD)-based [15], neural network (NN)-based [16], deep CNN-based [1,3], sparse representation (SR)-based and hybrid-based methods [18]. We will present the review of traditional and recent MIF methods.

The PCA-based fusion method is used in [30], which restores the spatial features and eliminates redundant information. Nonetheless, the fused image has spectral distortion that affects the overall quality of a fused image. The discrete wavelet transform-based fusion scheme is proposed in [15], which decomposes the images into low-frequency and high-frequency images. The discrete cosine transform (DCT)-based fusion scheme is utilized in [31], but the fused image has a lower spatial resolution. This method has the drawback of shift variance that results in an image with artifacts and noise. The hybrid DWT–PCA method is designed [32] to preserve spatial and spectral resolution. However, the fused image is distorted due to the shift-variance property. The discrete stationary wavelet transform is applied in [33] to address the shift-variance property issue and produce images with a better spectral resolution, but it still lacks spatial information. Tian et al. designed a combined mean-median-based hybrid DSWT-PCA approach in [34] that gives spatial and spectral details. Nevertheless, the output image has limited directional information; due to that, it fails to restore all salient features. The contour-let transform-based fusion approach is applied in [35] to address the issues [33]. However, Gibb’s effect is added in a fused image due to lack of shift-invariance. The amalgamated contour-let transform and PCA method along with contrast enhancement method is designed in [36]. This method combines the features of the spatial and spectral domains and preserves the contrast at the preprocessing step, but the fused image is distorted due to Gibb’s effect. To avoid Gibb’s phenomena, the non-sub-sampled contour-let transform (NSCT) is designed in [37]. Though this method achieves better fusion results, it is relatively time-consuming because it needs to use non-subsampled high pass filters due to the shift-invariance property. The authors in [38] applied a geometric algebra-based discrete cosine transform (GA-DCT) method for fusion of medical images. The source images are divided into various image blocks by the GA multi-vector form and then extend the DCT to GA space to introduce GA-DCT. Finally, they decompose images by the GA-DCT method to obtain the final fused image. Another approach for medical image fusion using guided filtering and image statistics in shearlet transform is designed in [39]. The images are decomposed into low and high frequency by shearlet transform. After that, the guided filter is used to obtain the weights of source images. These weights are added to the base layer to acquire a unified base layer. Finally, the guided filter and statistics fusion rule is used to fuse the coefficients.

Some good fusion results have been acquired in [40] by applying hybrid pulse code neural network (PCNN) and NSCT methods. Nonetheless, the image quality is degraded by how to tune the parameters and number of layers. Another approach is used [16] that concatenates the PCCN and focus-region level. The authors in [41] use non-subsampled contourlet transform (NSCT) and fuzzy local information clustering models to fuse the images. The authors employ log-ratio and mean-ratio operators to fuse two images. In [42], the authors have used amalgamated non-subsampled shearlet transform (NSST) using spatial frequency (SF) and PCNN. The NSST acts as a decomposition method while SF-PCNN fuses coefficients. Nonetheless, the PCNN has a large number of tuning parameters set as constant by the experience of humans that results in it only in multi-focus fusion methods.

A sparse representation (SR)-based scheme along with multi-scale decomposition method is proposed in [18] that inherits the drawbacks of SR and MSD. Another technique based on SR is presented by authors in [43] that classifies patches of an image from input images into various groups based on morphological similarities. The author in [27] introduced the MIF method that employs cartoon-texture decomposition (CTD) and uses the SR scheme to fuse decomposed coefficients. The authors in [44] have utilized the convolutional sparse representation (CSR)-based fusion method. The images are first decomposed into base and detail layers, and then a CSR-based scheme is applied to obtain the fused image. All aforementioned SR-based methods have either complicated fusion rules or distinguished SR algorithms. In addition, the dictionary training is cumbersome due to the dimension and number of dictionaries that lead to dimension disaster in image fusion (IF).

The CNN-based fusion methods have received a substantial breakthrough in the field of IF due to their unique and outstanding fusion results [45]. The authors in [46] have applied Res2net and double nonlocal attention models for the fusion of infrared and visible images. They used Res2net and dense connections into the encoder network with multiple receptive fields for extracting multiple features and used double nonlocal attention models as a fusion layer to design long-range dependencies for local features. Another approach to image fusion is implemented in [11], which uses ResNet-152. The low-frequency parts are fused by average weighted strategy while multi-layer features are extracted by high-frequency images using the ResNet-152 network. A CNN-based approach is designed in [47], which used a region of extraction scheme using morphology and the maximum difference rule. A MIF fusion using CNN and multi-scale decomposition method is designed in [48] to restore the textures and edges with good effect and a contrast pyramid, but it cannot address directional information and the blocking effect.

## 3. Proposed Methodology

**Figure 2:**
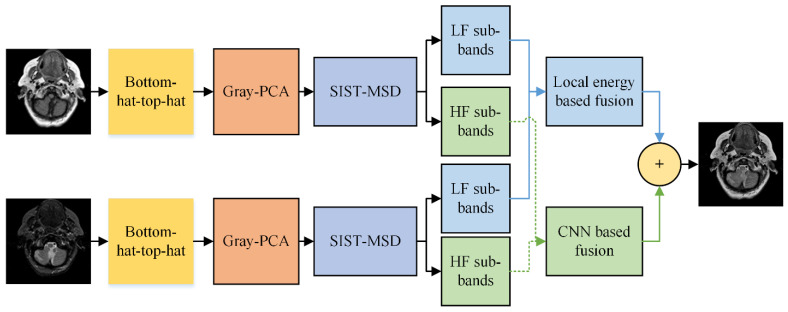
Proposed method.

Many recent image fusion (IF) methods have been designed to date, but the quality of the final fused image is not up to standard. Although CNN has achieved significant progress in the field of MIF, and its performance is more outstanding than recent fusion methods, CNN cannot be directly applied to a spatial domain that results in the loss of substantial features. Moreover, the multimodal medical images (MI) are affected due to non-uniform illumination and varying contrast, resulting in a distorted image with artifacts and noise. This paper proposes a novel hybrid fusion method that combats the aforementioned shortcoming and generates a final image containing all unique features with negligible loss of information. The proposed method uses bottom-hat–top-hat in combination with gray-PCA at the preprocessing stage, removing the non-uniform illumination and adjusting the contrast of an image. Additionally, the gray-PCA-based step converts three-channel gray images into one-channel grayscale images that help to preserve distinguishability between colors and textures more precisely. After that, the LSIST decomposition method decomposes the images into LF and HF bands to keep substantial features at various scales and directions with its fast computation. The LF images are fused by local energy that restores the whole energy information, while the HF bands are fed to two branches of CNN networks named the Siamese network that captures the smooth edges, sharp boundaries, and textures. Moreover, the CNN in the LSIST domain also addresses the issue of directly applying CNN to a spatial domain, which caused loss of information. The block diagram of the proposed method is presented in Figure 2.

We will discuss each step of the proposed method more precisely in sub-sections.

### 3.1. Morphological Bottom-Hat–Top-Hat Method

The multimodal medical images are affected due to non-uniform illumination and noise that distorts the image quality. The morphological bottom-hat–top-hat addresses the mentioned issues with its unique features. First, the bottom-hat–top-hat strategy is utilized on each medical image to evaluate the outcome of background noise. It is fact that medical images have intensity variation for background pixels due to non-uniform illumination, wherein the intensity of gray-level pixels is less than background pixels. Therefore, it is the prime task to eliminate the background lighting variation, which can be achieved by reducing the noise level. The noise level is reduced by applying the bottom-hat operation, which is computed by Equation (1):

(1) $$W_{b}(f)=(f•b)-f$$

The bullet sign represents the closing operation $W_{b}(f)$ on image f.

Equation (1) helps to observe the noise effect. The next step is to enhance the varying contrast of images, and it is computed by applying top-hat operation as given in Equation (2).

(2) $$W_{w}(f)=f-(f\circ b)$$

Here, the circle represents the opening operation $W_{w}(f)$ on image f.

Equation (2) eliminates the background noise by subtracting the bottom-hat from a top-hat image, producing an improved image.

After removing the noise from the image, we will apply gray-PCA that enhances the contrast of the image.

### 3.2. Gray-PCA

**Figure 3:**
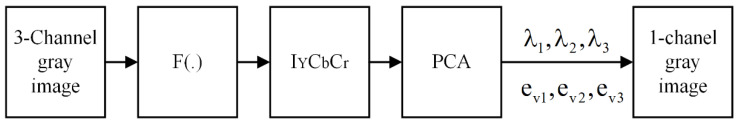
Schematic diagram of gray-PCA.

The gray-PCA is utilized in this work that helps to differentiate between textures and colors. The diagram of gray-PCA is depicted in Figure 3. Since it is known that grayscale images, especially medical images, preserve more information than RGB images, this helps to diagnose the disease more accurately. This can be achieved by applying gray-PCA that restores the distinguishability between colors and textures. The formation of a vectorized image ($I_{\mathrm{rgb}}\in R^{3}$) is the first step, which is achieved by three channels. After that, the separation of luminance and chrominance channels by utilizing transfer function f(.) is calculated by zero-mean of $YC_{b}C_{r}$. Subsequently, the Eigenvalues ($\lambda_{1}\geq \lambda_{2}\geq \lambda_{3}\in R^{1}$) and Eigen-vectors $e_{v1}\geq e_{v2}\geq e_{v3}\in R^{3}$ are calculated using PCA.

The obtained single-channel grayscale MI is determined by a linear amalgamation of three projections, wherein the weights are acquired by Eigen-vectors. Consequently, the first subspace projection provides multi-channel to single-channel gray mapping while the second and third projections restore the image details in the obtained single-channel gray image. The LSIST now processes the acquired images in the next section.

### 3.3. Local Shift-Invariant Shearlet Transform (LSIST)

The images obtained from preprocessing step are decomposed by a LSIST multi-scale decomposition (MSD) method that can effectively preserve the salient features at distinguished scales and directions. The LSIST scheme comprises two main stages: MSD and directional localization (DL). The MSD is obtained by applying non-subsampled pyramid filters (NSPF), while the DL is achieved by local small-scale shearing filters (SFs). This method is named LSIST since it utilizes the local SFs in shearing transform to enhance the effect of decomposition. At the same time, it is efficient in convolution computation in the time domain. These local SFs can remove blocking effects and reduce Gibb’s issue. The LSIST schemes can be framed by the following steps:

(a). Multi-scale decomposition (MSD): The non-subsampled pyramid filters with s scales are utilized that decompose images into LF and series of HF images that have the same size as input images. Let $f^{s}$ represents an image with s scales is decomposed into LF sub-band $f^{s+1}$ and series of HF images $g^{s+1}$ using NSPF.

(b). Directional localization (DL): This step involves the construction of a Meyer Window for high-frequency images $g^{s+1}$. The shearing filtering window W is produced in a pseudo polarization grid. Then, W from is aligned pseudo polarization into a Cartesian coordinate to acquire a new shearing filter $W_{\mathrm{new}}$. Subsequently, the 2D discrete fast Fourier transform (DFFT) is enumerated for $g^{s+1}$ to obtain a matrix $Fg^{s+1}$. The next step is to calculate various directional modules by employing band-pass filers to a matrix $Fg^{s+1}$. Afterward, the values of Cartesian samples are reconstructed and the inverse 2D-DFFT is used to generate the coefficients.

### 3.4. CNN for a Fusion of High-Frequency Bands

**Figure 4:**
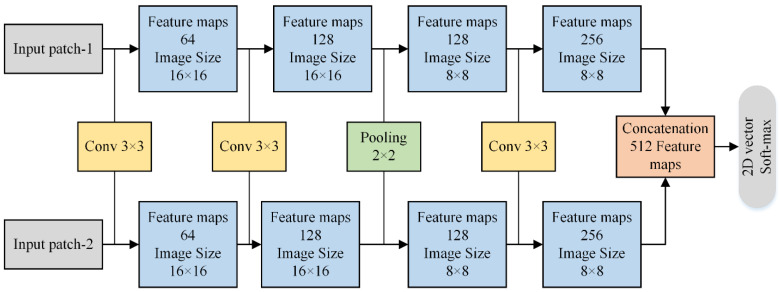
Two branches of Siamese network for HF sub-bands.

The CNN is employed in this paper to fuse HF images that can be depicted in Figure 4. The HF sub-bands from two images are fed to two branches of CNN that resemble weights and structures. This kind of network is called a Siamese network. The main purpose of using this network is to make classification easier because it trains two images simultaneously to allow the network to distinguish the two HF images.

Figure 4 shows the schematic diagram of two branches of the Siamese network. Each branch comprises three convolution layers (64, 128, and 256 filters, respectively) and one max-pooling layer. Each layer of convolution has 3×3 filter receptive with a stride of 1, wherein the max-pooling contains 2×2 with a step of 2. We have removed two fully connected layers in our method that can save the consumption of memory and computation time. In addition, it can also help to take images of any size instead of feeding fixed-size images. The 512 features maps obtained from concatenation are fed to a 2D vector soft-max layer that generates probability distribution for two classes. The stochastic gradient descent (SGD) soft-max loss function is applied for optimization purposes. Furthermore, the batch size is set to 128, with a weight decay of 0.00001 and momentum of 0.9.

**Figure 5:**
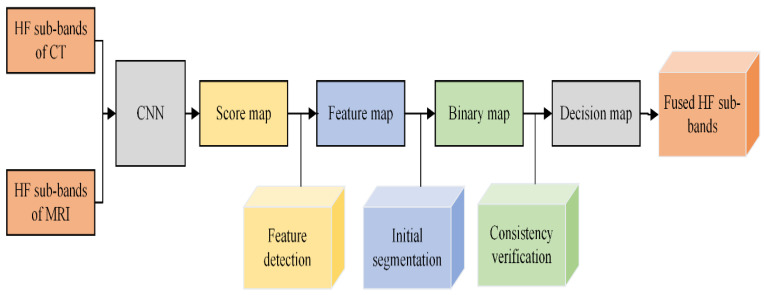
Block diagram of CNN Fusion for HF sub-bands.

The fusion of HF images using CNN consists of three steps: feature detection, initial segmentation, and consistency verification. We will discuss each sub-step in detail, and its diagram is presented in Figure 5.

(a). Feature detection: The HF sub-bands of two images ($C_{h}$ and $D_{h}$) are fed individually to two branches of a network to obtain a score map. Each coefficient in the score map indicates the feature characteristics. The weight of each coefficient ranges from 0 to 1, representing the pair of 16×16 corresponding blocks from two high-pass sub-images. Consequently, a feature map M of similar size is acquired from the score map by averaging the overlapping regions.

(b). Initial segmentation: After obtaining the feature map M, the next step is to obtain the binary map B. The choose-max selection strategy with a threshold of 0.5 is utilized to restore more useful features for producing binary maps. It is computed by Equation (3):

(3) $$B(x,y)=\left\{\begin{array}{l} 1,\;\;\;\;\;\;M(x,y)>0.5\\ 0,\;\;\;\;\;\;\mathrm{otherwise} \end{array}\right\}$$

Here, M indicates the feature map and B(x,y) represents the binary map. The obtained binary map B(x,y) is processed in the next step by consistency verification, and it is discussed in the next step.

(c). The consistency verification: The binary map may have some misclassified pixels whose values are not close to the surrounding pixel’s values, and this phenomenon is called singularity points. The consistency verification with an 8×8 window is applied in our scheme to remove the singularity points in the focus map.

Besides the singularity point issue, the artifacts are generated in the fused image during the decision map. The edge-preserving filtering method named guided filtering is applied to address the artifacts issue. The window size r and regularization parameter ε need to be assigned, and we have chosen 4 and 0.1 values for r and ε, respectively. Finally, the resultant fused image $F_{h}$ for HF sub-bands is computed by Equation (4).

(4) $$F_{h}=D_{m}(x,y)C_{h}(x,y)+(1-D_{m}(x,y))D_{h}(x,y)$$

here, $D_{m}(x,y)$ represents the decision map for the image, and $C_{h}(x,y)$ and $D_{h}(x,y)$ indicate the two HF sub-band images, respectively.

### 3.5. Fusion of Low-Frequency Images by Local Energy Method

The LF images contain the energy information, and whole energy details can be retained by the proposed fusion method. The local energy En(x,y) for the LF sub-band $L_{j}$ can be obtained by Equation (5):

(5) $$En(x,y)=\sum_{m}\sum_{n}L_{j}{\left(x+m,y+n\right)}^{2}W_{c}(m,n)$$

The $W_{c}$ indicates template size of 3×3, while m and n represent the size of the local area. After computing the En(x,y), the next step is to find the salience factor $S_{j}$, calculated by Equation (6):

(6) $$S_{j}^{CD}(x,y)=\frac{2\sum_{m}\sum_{n}L_{j}^{C}(x+m,y+n)L_{j}^{D}(x+m,y+n)}{En^{C}(x,y)+En^{D}(x,y)}$$

here, $S_{j}$ represents the salience factor, $L_{j}^{C},\;L_{j}^{D}$ indicates coefficients for LF sub-bands of images C and D.

The purpose of calculating $S_{j}$ is to choose whether averaging or selection mode is utilized for a fusion of LF sub-bands. We set a threshold T for it, and its value is set to 0.5. The averaging mode is employed if $S_{j}^{CD}>T$ while selection mode will be used if $S_{j}^{CD}\leq T$.

The averaging mode for final fused LF sub-bands $L_{j}^{F}$ is calculated by Equation (7):

(7) $$L_{j}^{F}(x,y)=\alpha_{C}\cdot L_{j}^{C}(x,y)+\alpha_{D}\cdot L_{j}^{D}(x,y)$$

The $\alpha_{C}$ and $\alpha_{D}$ indicate the weights of LF sub-bands C and D.

The $\alpha_{C}$ and $\alpha_{D}$ can be obtained by Equations (8) and (9):

(8) $$\alpha_{C}=\left\{ \begin{array}{l} \alpha_{\min}\;\;\mathrm{for}\;\;En_{l}^{C}(x,y)<En_{l}^{D}(x,y)\\ \alpha_{\max}\;\mathrm{for}\;\;En_{l}^{C}(x,y)\geq En_{l}^{D}(x,y) \end{array}\right.$$

(9) $$\alpha_{D}=1-\alpha_{C}$$

Subsequently, the selection mode is computed when $S_{j}^{CD}\leq T$ using Equation (10):

(10) $$\alpha_{j}^{F}=\left\{ \begin{array}{l} \alpha_{j}^{C}(x,y)\;\;\;\;\mathrm{for}\;\;En_{l}^{C}(x,y)\geq En_{l}^{D}(x,y)\\ \alpha_{j}^{D}(x,y)\;\;\;\;\mathrm{for}\;\;En_{l}^{C}(x,y)<En_{l}^{D}(x,y) \end{array}\right.$$

It can be mathematically pointed out by Equation (10) that only those coefficients will be chosen that have high energy while the coefficients that have less energy values would be discarded.

### 3.6. Reconstruction

The obtained fused images from LF and HF sub-bands are reconstructed by linear superposition using Equation (11).

(11) F(x,y)=C(x,y)+D(x,y)

here, F(x,y), C(x,y) and D(x,y) represent the final fused image and fused image from LF and HF sub-bands, respectively.

## 4. Simulation Results, Datasets, and Evaluation of Fusion Methods

**Figure 6:**
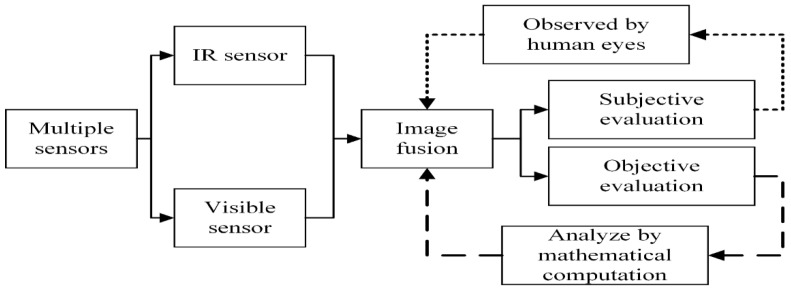
The process of evaluating the subjective and objective evaluation.

The experiments are performed on CT and MRI medical images to validate the superiority of the proposed method over conventional and recent fusion methods. The CT and MRI images are taken from [49,50,51]. The proposed method is compared with DCT [31], DWT [15], NSST-SF-PCNN, CSR [44], and CNN [48]. We have compared the simulation results on subjective and objective quality parameters. The process for evaluating the subjective and objective analysis is shown in Figure 6.

The subjective evaluation is used to evaluate the quality of fused images according to human perception, such as brightness, contrast, edges, contours, noise, and so on. Therefore, the researchers in image fusion evaluate the subjective evaluation through experts’ experience by the human visual system (HVS). In this paper, we have shown fusion results to twelve field experts who evaluated the quality of the fused method by their visual perception experience. To further justify the supremacy of fusion methods, we have used the quality transfer of edges from source images to fused image ($Q_{F}^{AB}$) [4], correlation (CRR) [52], average gradient (AG) [52], and noise ratio from source images to fused image ($N_{F}^{AB}$) [53] as an objective evaluation. The higher values of $Q_{F}^{AB}$, CRR, and AG correspond to better quality fused images with essential features. We have performed simulations results on MATLAB 2016b on a core i5 3.2 GHz microprocessor with RAM of 8 GB. We have set the default setting of all comparing methods. We have taken RGB images that are visualized as gray images.

**Figure 7:**
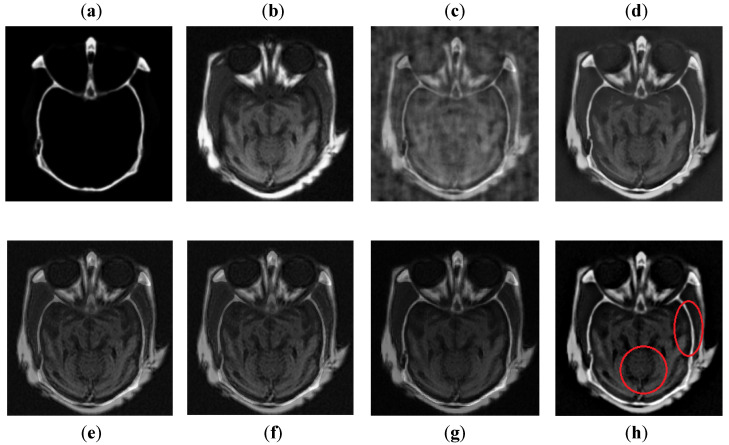
Data-1: (**a**) CT image, (**b**) MRI image, (**c**) DCT, (**d**) DWT, (**e**) NSST-SFT-PCNN, (**f**) CSR, (**g**) CNN, and (**h**) Proposed.

It can be observed in Figure 7 that images obtained by DCT have more noise and artifacts that distort the quality of a final image. The DWT method produces less noise than DCT, but the overall image is blurred, which affects the quality of the image. The NSST-SF-PCNN and CSR have better visual effects and produce better quality, but the image is still blurred, and some information is lost. The CNN method produces better results, and the image is more vivid and smooth than other existing methods, but its contrast and sharpness of the edges are still not up to the mark. It can be analyzed that the proposed method acquires better results than all mentioned fusion methods. The fused image has very negligible noise with better contrast. The edges are sharper with smoother boundaries. Hence, the final image has better visual effects with more salient information.

**Figure 8:**
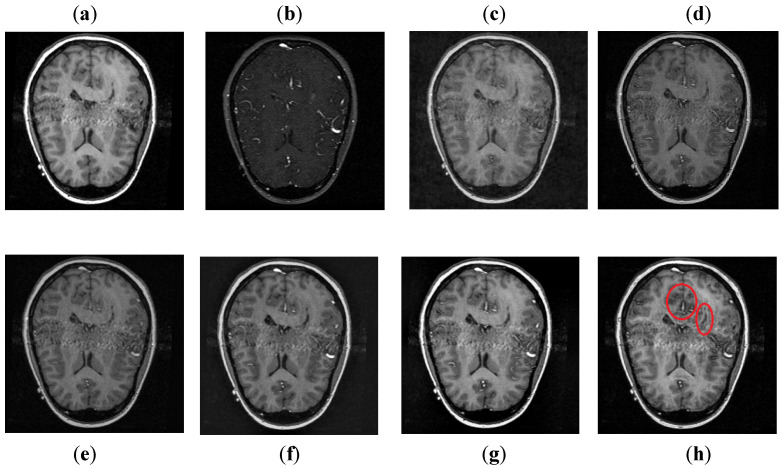
Data-2: (**a**) CT image, (**b**) MRI image, (**c**) DCT, (**d**) DWT, (**e**) NSST-SFT-PCNN, (**f**) CSR, (**g**) CNN, and (**h**) Proposed.

It can be depicted in Figure 8 that the DCT method contains most of the information from the CT image, but it fails to obtain more information from the MRI image. The DWT and NSST-SF-PCNN produce images with good contrast, but both methods cannot obtain more information about edges. The CSR method has better results than DCT, DWT, and NSST-SF-PCNN by producing less noise and good contrast images. However, this method also fails to capture more information about soft tissues. The CNN scheme and proposed method produce better results with uniform illumination, but it can be observed in red boxes that the proposed method can capture more information about edges and soft tissues and preserve whole useful information. Additionally, the proposed method effectively retains the complete information from both source images.

**Figure 9:**
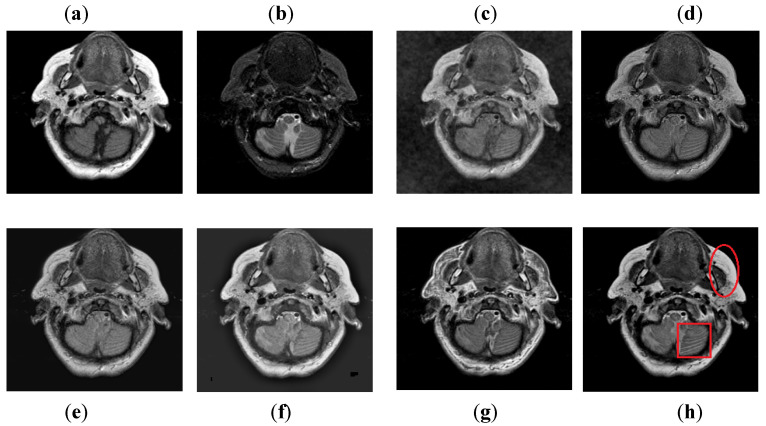
Data-3: (**a**) CT image, (**b**) MRI image, (**c**) DCT, (**d**) DWT, (**e**) NSST-SFT-PCNN, (**f**) CSR, (**g**) CNN, and (**h**) Proposed.

It can be visually observed in Figure 9 that the DCT method has more artifacts that distort the overall quality of a fused image. The DWT and NSST-SF-PCNN produce almost similar results, but DWT has more contrast and less noise than NSST-SF-PCNN. However, both methods have poor detailed and structured information that significantly loses valuable information about soft tissues. The CSR method effectively captures more information from the CT image, but due to non-uniform illumination, it drastically fails to restore more information about soft tissues from MRI image. On the other hand, the CNN method retains most of the information about soft tissues from an MRI image with good contrast, but it cannot completely capture edges and boundaries from a CT image that affect the image quality. The proposed method shows its superiority over other methods by producing a fused image that contains information from both source images. It has better contrast with very negligible artifacts. Additionally, it can be further explored from red boxes that the proposed method can effectively retain the soft tissues from MRI image and preserve bright and sharp detail about bones from a CT image.

**Figure 10:**
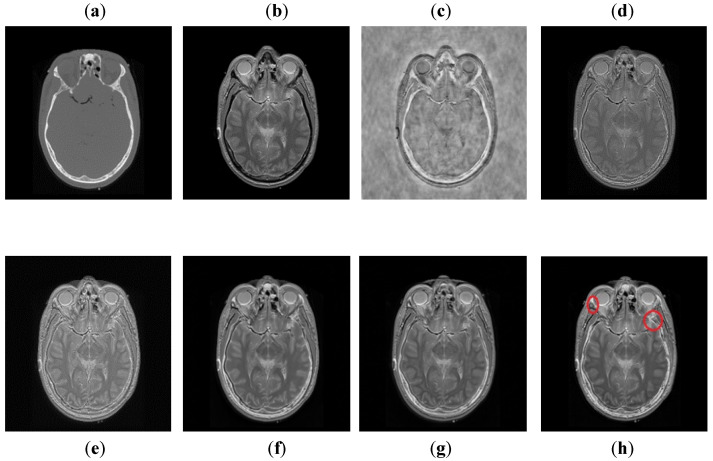
Data-4: (**a**) CT image, (**b**) MRI image, (**c**) DCT, (**d**) DWT, (**e**) NSST-SFT-PCNN, (**f**) CSR, (**g**) CNN, (**h**) and Proposed.

It can be seen in Figure 10 that DCT has poor results with more artifacts. The DWT method also has artifacts and pixel discontinuities due to limited directional information. The image obtained by NSST-SF-PCNN has better visual effects, but it has uneven illumination, and due to that, the fused image lost some vital information. The CSR and CNN methods resemble each other, but the contrast of CNN is better than CSR. Both methods acquire good visual results and have good image contrast, but some edges and boundaries are not vivid. The proposed method again shows its supremacy by producing output image with vivid contrast and high resolution. Additionally, it can be depicted in red boxed to capture smooth edges and contours more precisely.

**Figure 11:**
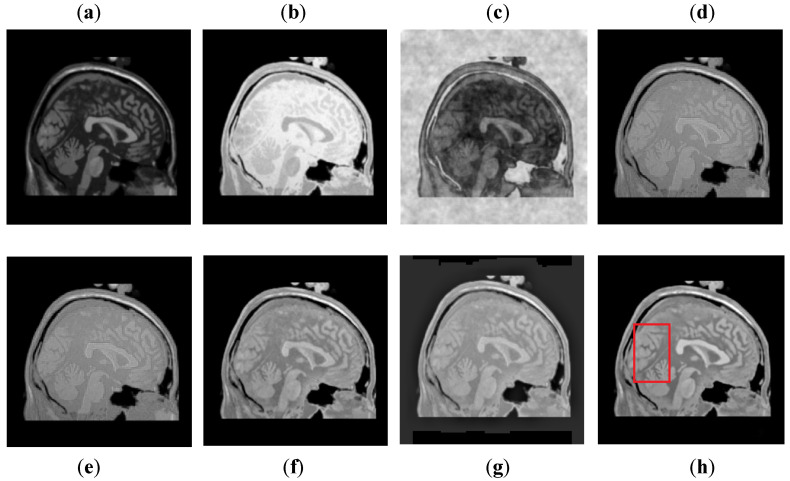
Data-5: (**a**) CT image, (**b**) MRI image, (**c**) DCT, (**d**) DWT, (**e**) NSST-SFT-PCNN, (**f**) CSR, (**g**) CNN, and (**h**) Proposed.

It can be concluded from Figure 11 that the DCT method has more block artifacts, whereas the DWT and NSST-SF-PCNN acquire almost the same information. Both images are blurred with poor contrast. Additionally, these methods have jagged contours and blurred images, which shows the limitation of algorithms. The CSR and CNN methods produce images with good detailed information with fewer artifacts, but the CSR method retains better patterns and textures than the CNN method with good contrast. The image obtained by the proposed method has even more precise patterns and textures than the CSR method. In addition, the proposed image is more vivid with bright contrast that helps to preserve all detailed information effectively.

**Figure 12:**
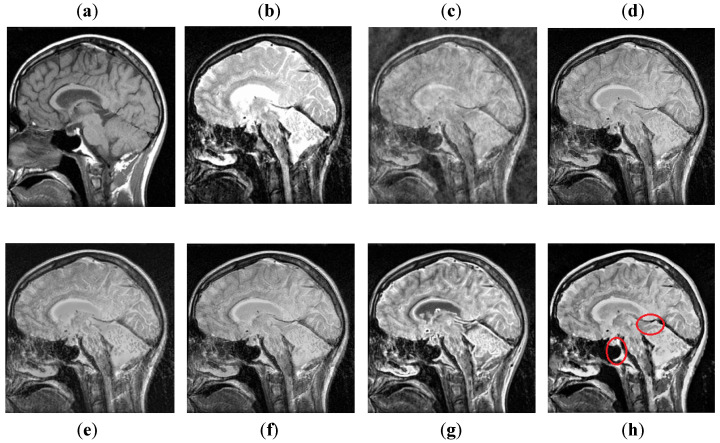
Data-6: (**a**) CT image, (**b**) MRI image, (**c**) DCT, (**d**) DWT, (**e**) NSST-SFT-PCNN, (**f**) CSR, (**g**) CNN, and (**h**) Proposed.

The DCT method in Figure 12 has poor fusion results due to blocking artifacts and noise. The DWT method has less noise than DCT, but it has non-uniform illumination that leads to loss of information about edges. The NSST-SF-PCNN and CSR methods provide sufficient spatial details and provide good soft tissue information. Nonetheless, the edges and textures are not smooth, and the image is not vivid. The CNN method helps to capture more information about edges. However, the MRI image is more dominant during the fusion process; the CNN method lost some significant information from the CT image. The proposed method again wins the competition by producing unique fusion results. It retains information from both source images with uniform contrast and less artifacts. Furthermore, it can be analyzed in red boxes that the proposed method effectively restores sharp edges and contours with vivid high resolution.

### 4.1. User Case

We conducted a user study in which twelve field specialists were asked to rank each method on six pairs of source images. The following is a more in-depth discussion:

To demonstrate the supremacy of our method over state-of-the-art methods, we conducted a user case study on six datasets. We requested twelve experts to rank the results of all approaches based on their preferences. For each pair of source images, each expert was asked to score six results (the proposed work and five other methods) on a scale of one point (least favored) to six points (most preferred). Experts on the following four points carry out subjective analysis: (1) precise details, (2) image contrast, (3) noise in the fused image, and (4) no loss of information. The experts were given anonymized results in a random order to avoid subjective bias.

Furthermore, the user study was conducted in the same scenario (room, monitor, and light). After the experts graded all of the simulation results, we assessed the average points gained by each approach on six pairs of source images. The average points scored by experts using each approach on six pairs of source photos are given in Table 1.

**Figure 13:**
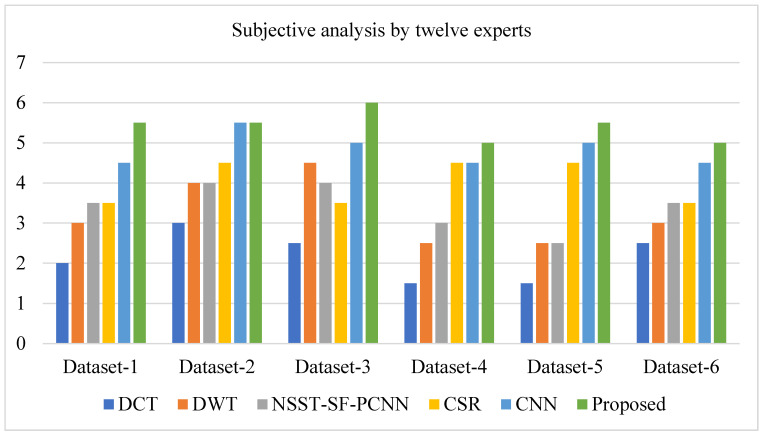
A user case study (average points obtained by experts on each method).

It can be depicted from Figure 13 that the proposed method acquires more points for each dataset (except for dataset-2, where the proposed method and CNN have the same score) by experts’ visual experience.

Table 2 presents the average values of eight pairs of medical images for comparison based on objective evaluation assessments. Due to the space limit, we have shown the average values for each quality parameter. The higher values are written in bold letters in Table 2 for the reader’s ease. In contrast, the small value for $N_{F}^{AB}$ refers to less artifacts and noise in the fused image.

**Figure 14:**
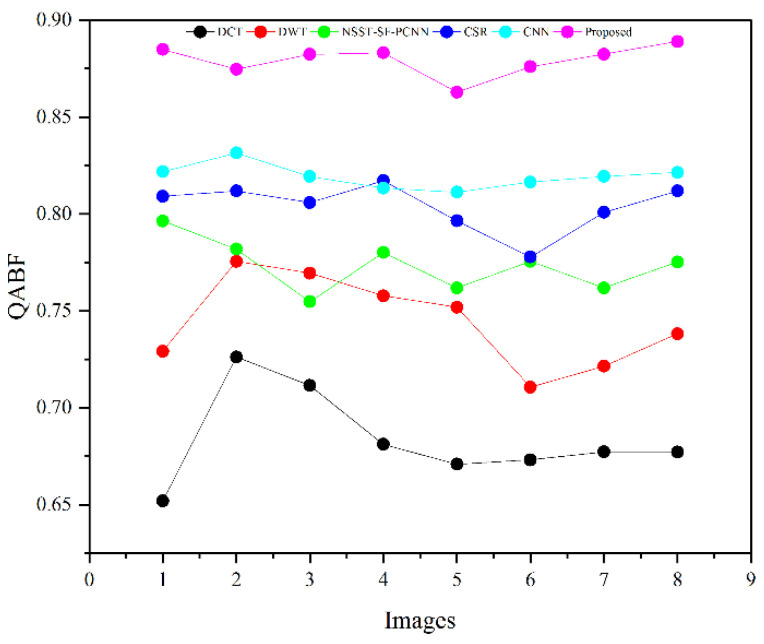
Plotting for eight pairs of images.

**Figure 15:**
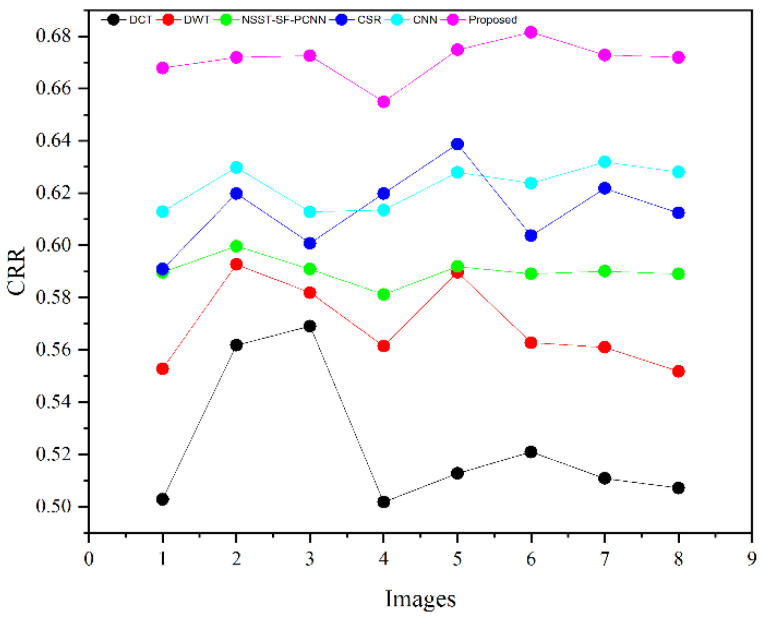
CRR plotting for eight pairs of images.

**Figure 16:**
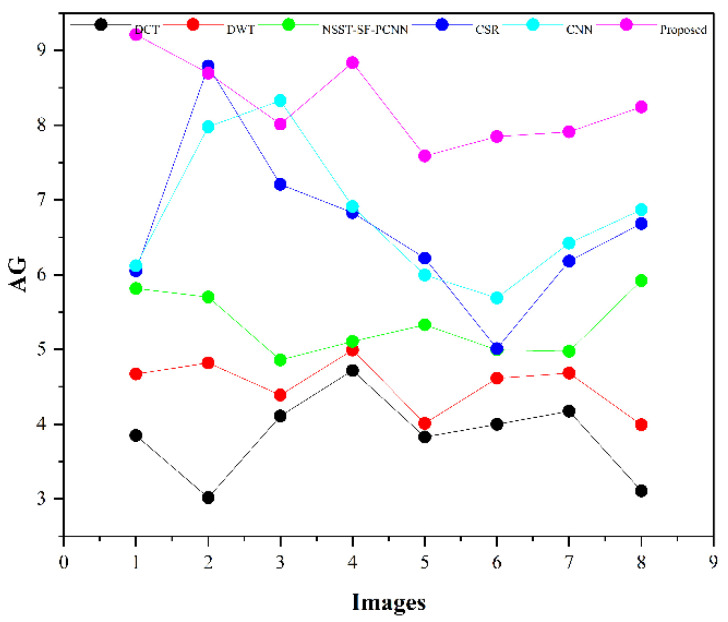
AG plotting for eight pairs of images.

**Figure 17:**
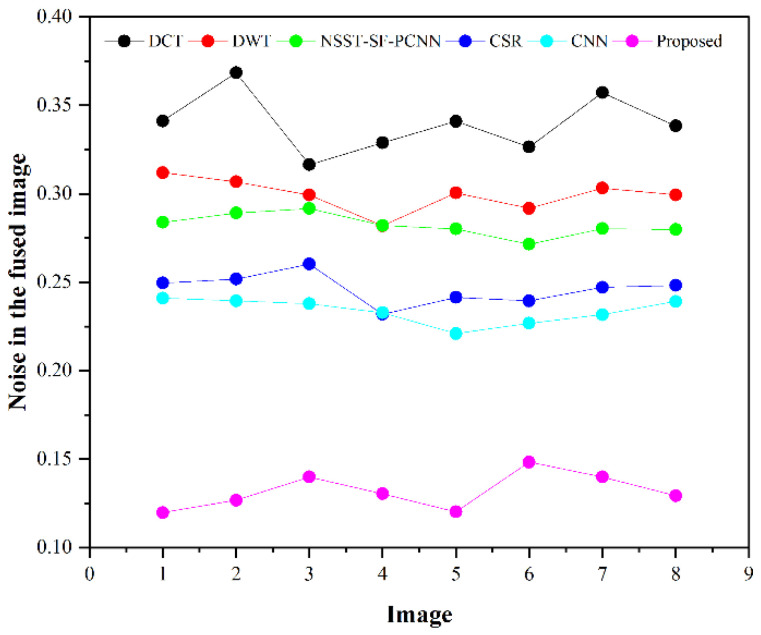
Noise plotting for eight pairs of images.

We have also plotted all methods for each parameter in Figure 14, Figure 15, Figure 16 and Figure 17, respectively. The higher values for $Q_{F}^{AB}$, CRR, and AG correspond to images with more features, structure, and similarity between source images and the fused image. On the contrary, the lower value of $N_{F}^{AB}$ refers to an image with less artifacts and noise. The higher values for $Q_{F}^{AB}$, CRR, AG, and lower values of $N_{F}^{AB}$ are highlighted by bold letters for the reader’s feasibility. It can be depicted from Table 2, Figure 14, Figure 15 and Figure 16 that small average values for $Q_{F}^{AB}$, CRR, AG, and higher values of $N_{F}^{AB}$ (Figure 17) show the deficiency of the DCT method. On the other hand, the DWT produces better results than DCT, but it still fails to capture significant features, structures, and contrasts. The NSST-SF-PCNN acquires intermediate results, and it has a better ability to restore features, but their results are not up to the mark. The values obtained by CSR and CNN are better than other methods, revealing that they can preserve more features, contours, and edges information with better contrast.

Compared with all other methods, the proposed method effectively retains more structure, features, and contrast, which shows its superiority. In addition, the lower values for $N_{F}^{AB}$ proves that the proposed method has unique characteristics of removing noise. In a nutshell, the proposed method shows its superiority on objective parameters over other fusion methods. The CNN stands in the second number by producing better results than DCT, DWT, NSST-SF-PCNN, and CSR.

### 4.2. Computation Time

The average computation time for the proposed method and the other state-of-art fusion methods is presented in Table 3. The simulations are performed for computation time (t) in seconds on MATLAB 2016b with a corei5 3.20 GHz processor and 8 GB RAM. It can be depicted from Table 3 that the CSR method has the longest running time, which shows its poorest timelines. The DWT method has the fastest running time; however, its performance is poor on subjective and objective evaluation. The computation time of the proposed method is longer than DCT and DWT, but better visual effects can sacrifice for timelines. Furthermore, the computation time for the proposed method is shorter than NSST-SF-PCNN, CSR, and CNN, while it also produces better visual effects. Therefore, the computation time for the proposed method is acceptable.

### 4.3. Comparison Discussion

We have performed experiments on eight pairs of medical images, but we have shown simulation results of only six pairs of medical images due to the space limit. Figure 7, Figure 8, Figure 9, Figure 10, Figure 11 and Figure 12 present the results of the proposed method and five recent fusion methods. It can be seen that the DCT method has poorer performance than all other methods due to noise and artifacts. The DWT method provides good detailed information, but the overall image is blurred, and it also has artifacts due to limited directional information that distort the quality of a fused image. The NSST-SF-PCNN method provides sufficient spatial details and gives good information about soft tissues. Nonetheless, the edges and textures are not smooth, and the image is not vivid. The quality of the CSR and CNN method is better than DCT, DWT, and NSST-SF-PCCN by producing good texture and edges information with less artifacts and noise. The fusion results of CSR and CNN methods resemble a little bit, but the CNN method has even more brightness and contrast that produce more detailed information. It can be analyzed that the proposed method gives outstanding results than all aforementioned methods. The fused image has bright contrast with negligible noise due to preprocessing noise removal and contrast enhancement scheme features. Additionally, the proposed method restores all notable features due to the attractive LSIST property that can capture details at various scales and directions. Furthermore, the proposed method has sharp edges and smooth contours with complete energy information due to CNN and local fusion energy rules. Therefore, it can be concluded by a human visual system that the proposed method shows its efficacy more than the compared fusion methods due to its outstanding fusion results. Furthermore, the proposed method obtains outstanding values compared to other methods for $Q_{F}^{AB}$, CRR, AG, and $N_{F}^{AB}$ that can be seen in Table 2, Figure 14, Figure 15, Figure 16 and Figure 17, respectively. Based on subjective and objective analysis, it can be summarized that the proposed method has more potential to retain significant features such as smooth edges, sharp boundaries, and contours with better contrast and very negligible noise. This not only helps doctors to save their time and energy by obtaining all substantial information from one image but also helps to diagnose the disease accurately.

Although the proposed method achieves superior performance for MIF, it still needs to be improved in terms of computational time and applicability in other fields. This work can be extended to general image fusion tasks such as multi-focus, whereas employing transfer learning in CNN can help minimize memory usage and save computational time.

## 5. Conclusions

This work presents a novel hybrid MIF method that amalgamates the advantages of each proposed step, including bottom-hat–top-hat along with gray-PCA, LSIST, and CNN to acquire a high-quality fused image. In this work, the bottom-hat–top-hat preprocessing scheme is used to remove the non-uniform illumination and noise. Then, grey-PCA is used for conversion of RGB image to gray image, which preserves substantial features by increasing the contrast of an image. In addition, this step eliminates the redundant amount of data with its robust operation. The LSIST is applied to processed images that decompose them into LP and HP sub-bands using non-subsampled pyramid filters (NSPF) and shearing filters (SFs) that further enhance the image quality by retaining the significant features in various directions and scales. Then, the two branches of Siamese CNNs are used for HP sub-bands, which produce sharp edges and textures whilst removing the artifacts. After that, the local energy fusion scheme using average and selection mode is used for LP sub-bands that restore the energy information. Finally, an inverse transformation is deployed to fuse a final image containing enriched details and substantial features with negligible artifacts. The simulation results reveal that the proposed work generates a visually higher-quality image than conventional fusion methods on subjective evaluation assessment, which is verified by twelve field specialists in user case study. Additionally, it can be analyzed that the proposed method achieves 0.6836 to 0.8794, 0.5234 to 0.6710, and 3.8501 to 8.7937 gain for $Q_{F}^{\mathrm{AB}}$, CRR, and AG parameters, respectively, compared to existing methods. At the same time, the noise reduction from 0.3397 to 0.1209 further justifies the superiority of the proposed method over other state-of-art fusion methods.

## Figures and Tables

**Table 1:** The average points scored by experts using each approach on six pairs of source photos.

Dataset-1	DCT	DWT	NSST-SF-PCNN	CSR	CNN	Proposed
1	2	3	3.5	3.5	4.5	5.5
2	3	4	4	4.5	5.5	5.5
3	2.5	4.5	4	3.5	5	6
4	1.5	2.5	3	4.5	4.5	5
5	1.5	2.5	2.5	4.5	5	5.5
6	2.5	3	3.5	3.5	4.5	5

**Table 2:** The average values for objective evaluation parameters for fusion methods.

Method	DCT	DWT	NSST-SF-PCNN	CSR	CNN	Proposed
$Q_{F}^{AB}$	0.6836	0.7443	0.7735	0.8039	0.8193	**0.8794**
CRR	0.5234	0.5692	0.5902	0.6134	0.6225	**0.6710**
AG	3.8501	4.5213	5.3277	6.6212	6.7895	**8.7937**
$N_{F}^{AB}$	0.3397	0.2993	0.2823	0.2462	0.2337	**0.1209**

**Table 3:** The average running time for proposed method and other methods (unit: seconds).

Method	DCT	DWT	NSST-SF-PCNN	CSR	CNN	Proposed
Time	11.7	10.2	36.7	101	18.7	11.3

## Data Availability

The data that supports the finding of this research is available in this paper.
